# Virulence of Two Entomophthoralean Fungi, *Pandora neoaphidis* and *Entomophthora planchoniana*, to Their Conspecific (*Sitobion avenae*) and Heterospecific (*Rhopalosiphum padi*) Aphid Hosts

**DOI:** 10.3390/insects10020054

**Published:** 2019-02-13

**Authors:** Ibtissem Ben Fekih, Annette Bruun Jensen, Sonia Boukhris-Bouhachem, Gabor Pozsgai, Salah Rezgui, Christopher Rensing, Jørgen Eilenberg

**Affiliations:** 1 Plant Protection Laboratory, National Institute of Agricultural Research of Tunisia, Rue Hédi Karray, 2049 Ariana, Tunisia; soribou@yahoo.fr; 2 Department of Plant and Environmental Sciences, Faculty of Science, University of Copenhagen, Thorvaldsensvej 40, 3rd floor, 1871 Frederiksberg C, Denmark; abj@plen.ku.dk (A.B.J.); jei@plen.ku.dk (J.E.); 3 Institute of Environmental Microbiology, College of Resources and Environment, Fujian Agriculture and Forestry University, Fuzhou 350002, China; rensing@fafu.edu.cn; 4 State Key Laboratory of Ecological Pest Control for Fujian and Taiwan Crops, Fujian Agriculture and Forestry University, Fuzhou 350002, China; 5 Institute of Applied Ecology, Fujian Agriculture and Forestry University, Fuzhou 350002, China; 6 Department of ABV, National Agronomic Institute of Tunisia, 43 Avenue Charles Nicolle, 1082 EL Menzah, Tunisia; salahrezgui@yahoo.fr

**Keywords:** aphids, *Pandora neoaphidis*, *Entomophthora planchoniana*, virulence

## Abstract

*Pandora neoaphidis* and *Entomophthora planchoniana* (phylum Entomophthoromycota) are important fungal pathogens on cereal aphids, *Sitobion avenae* and *Rhopalosiphum padi*. Here, we evaluated and compared for the first time the virulence of these two fungi, both produced in *S. avenae* cadavers, against the two aphid species subjected to the same exposure. Two laboratory bioassays were carried out using a method imitating entomophthoralean transmission in the field. Healthy colonies of the two aphid species were exposed to the same conidial shower of *P. neoaphidis* or *E. planchoniana*, in both cases from a cadaver of *S. avenae*. The experiments were performed under LD 18:6 h at 21 °C and a successful transmission was monitored for a period of nine days after initial exposure. Susceptibility of both *S. avenae* and *R. padi* to fungal infection showed a sigmoid trend. The fitted nonlinear model showed that the conspecific host, *S. avenae*, was more susceptible to *E. planchoniana* infection than the heterospecific host *R. padi*, was. In the case of *P. neoaphidis*, LT_50_ for *S. avenae* was 5.0 days compared to 5.9 days for *R. padi*. For *E. planchoniana*, the LT_50_ for *S. avenae* was 4.9 days, while the measured infection level in *R. padi* was always below 50 percent. Our results suggest that transmission from conspecific aphid host to heterospecific aphid host can occur in the field, but with expected highest transmission success to the conspecific host.

## 1. Introduction

*Sitobion avenae* (Fabricius, 1775) and *Rhopalosiphum padi* (Linnaeus, 1758) aphids are serious pests, commonly coexisting in cereal fields [1,2,3]. Both species can cause economic damage through their feeding activity and their role in the dissemination of phytoviruses, such as the Barley Yellow Dwarf Virus (BYDV) [3]. Entomophthoralean fungi such as *Pandora neoaphidis* (Remaudière et Hennebert) and *Entomophthora planchoniana* Cornu are obligate aphid pathogens [4,5,6,7] and can cause epizootics among aphids in cereals [4,8,9,10,11]. It is however not known to which extent infection can be transmitted between *S. avenae* and *R. padi* in the field and if an epizootic in one of these aphid species can result in infection in the other aphid species.

To understand the transmission of a fungal pathogen between aphid host species occurring in the same crop, we need comparative virulence bioassay studies mimicking the situation occurring in the field. Virulence studies with hypocrealean insect pathogenic fungi, such as species from the genera *Beauveria* and *Metarhizium*, can be done by subjecting the target insect to suspensions with a predefined, known concentration of conidia. This is possible because these hypocrealean fungi have small, dry conidia which can readily be dissolved in water supplemented with a detergent [12,13]. Entomophthoralean fungi, however, have large, sticky conidia which are actively discharged from dead insects before landing on the cuticle of an uninfected individual [14,15,16,17]. These conidia cannot be dissolved in water due to their sticky nature, so assays with predefined concentrations are technically not feasible.

The main methods for performing bioassays of entomophthoralean fungi have therefore to focus on alternatives. Insects are placed in small plastic cups and subjected to conidia discharged from insect cadavers or from mycelial mats produced in vitro [13,18,19,20,21,22]. Measuring the conidia concentrations can, to some extent, be done by adding cover slips in the cups during exposure and afterwards counting conidia on these cover slips [13,22,23]. Such methods have the general drawback that conidia concentrations cannot be precisely predefined, so exposing replicates to the exact same conidia concentrations is almost impossible to achieve. A main challenge in comparative studies on the virulence of entomophthoralean fungi to different hosts is therefore to apply a method that allows comparisons between replicates. Besides, of course, sample size and replicates should be sufficient to allow comparisons. 

Our aim was to tackle this issue and to study the virulence of the two entomophthoralean fungi *P. neoaphidis* and *E. planchoniana* (both produced in *S. avenae*) against the two aphid species *S. avenae* and *R. padi* when exposed to the same conidial exposure. Our hypothesis was that the virulence against conspecific and heterospecific hosts differs, and we predicted a higher virulence against the conspecific host.

## 2. Materials and Methods

### 2.1. Rearing of Target Aphids

*S. avenae* and *R. padi* were originally provided on barley banker plants from EWH BioProduction ApS (Tappernøje, Denmark). Colonies of both aphid species were transferred to wheat plant and maintained separately in ventilated plexiglass cages (0.60 m × 0.30 m × 0.30 m) at 21 °C. Boxes with fresh wheat plants (cultivar Dacanto) were provided weekly to maintain the rearing of target aphids.

### 2.2. Sampling and Inoculum Preparation

Barley leaves and inflorescences infested with *S. avenae* were sampled at Bakkegaarden, an experimental agricultural field in Taastrup belonging to University of Copenhagen. Samples were kept in ventilated boxes and brought to the laboratory for microscope examination. Living apterous adults of *S. avenae* were picked up and incubated individually in small sterilized plastic cups (30 mL) containing 1.5% water–agar. Pieces of wheat leaves secured in the water–agar served as a food source for the aphids and the cups were maintained in an incubator at 21 °C under LD 18:6. Aphids were checked daily for cases of entomophthoralean infection. The investigation of new cases of mycosis was performed for 7 days, the time required for the development of fungus in the suspected infected aphids. Once dead, *S. avenae* cadavers, both with or without external signs of fungal structures, were carefully picked up from the wheat leaves and placed for incubation in a humid chamber on the top of a 15 mm × 15 mm cover slip for 12–24 h at 21 °C to promote conidiophores development and thus conidia discharge. The first projected conidia from each cadaver were mounted in lactic acid and used for identification of fungus species using phase-interference on an Olympus AX70 Provis light microscope at 400× magnification and related keys [24]. Sterile fine forceps were used during the manipulation to avoid cross contamination. Only *S. avenae* cadavers with good sporulation of either *P. neoaphidis* or *E. planchoniana* were used in the bioassay.

### 2.3. Bioassay Setup

**Figure 1:**
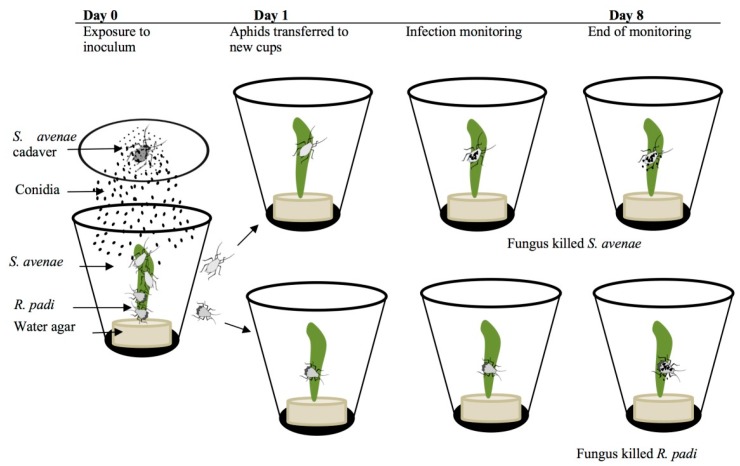
Inoculation and monitoring procedure of the aphids *Sitobion avenae* and *Rhopalosiphum padi* exposed to the fungi *Pandora neoaphidis* or *Entomophthora planchoniana* using the conidia shower method. Ten specimens of each of the two aphid species were infected in the same cup simultaneously to ensure they were exposed to same conidia concentration.

For the transmission, we established an environment mimicking field situations by introducing both aphid species in the same inoculation cups with small wheat leaves as food source. The bioassay procedure was based on descriptions of entomophthoralean bioassays [22]. Ten apterous young adults of *S. avenae* and ten *R. padi* were transferred jointly onto fresh wheat leaves in each cup. The two main reasons for doing so are these: First, that by being together, the two aphid species in each cup were allowed to place themselves on the leaves according to their habitat preferences, and second, afterward they were subjected to the same conidial shower as in real field conditions. A freshly sporulating cadaver of *S. avenae* was attached with Vaseline^®^ onto the inner side of a lid and placed over the ten *S. avenae* and ten *R. padi* (Figure 1). Controls included the same number of aphids just without the sporulating aphid cadaver. Five replications, each using new cadavers, were performed for each of the two fungus treatments (*E. planchoniana* and *P. neoaphidis*, respectively). Healthy aphids were exposed to conidial showers for six hours in a humid chamber. Then, the inoculum was removed, and the cups were incubated at 21 °C under LD 18:6 h. Twenty-four hours from the exposure, treated aphids were transferred into individual cups to avoid cross-infection between aphid specimens. Fresh wheat leaves were provided for each aphid and incubation was conducted under the same conditions as above. Incubated aphids were inspected daily for mycosis for eight days after exposure. Each day, dead aphids were collected and checked for the presence of fungal structures (conidia, conidiophores or rhizoids) using a dissecting light microscope and were later incubated in a humid chamber for 12–24 h at 21 °C. After incubation, aphid cadavers with no external signs of infection were dissected and stained with lactic acid to look for the presence of resting spores. In total, 50 *S. avenae* and 50 *R. padi* were exposed to fungal treatments. During the study, dead aphids with no observed signs of fungal infection were also recorded.

### 2.4. Data Analysis

Overall mortalities, with or without observed sign of fungal infection, were counted and arcsine transformed, following the formula [Arcsin ((√×)/100)] to standardize the data distribution and stabilize the variance [25]. Repeated measures ANOVA was used to test whether mortalities after fungal treatments were different from those registered within control.

Afterward, only aphids showing clear fungal structures were considered as mortality counts in the analysis. Cumulative mortality percentages for each day of incubation were also arcsine transformed. A non-linear least-square regression model was fitted on the time-mortality data of the aphids from each fungus infection (M), using the nls () function in R statistical software [26] with the implemented Gauss-Newton algorithm. Packages “data.frame” [27] and “investr” [28] were used to facilitate the analysis. The adopted model is expressed as M (ij) = k/(1 + exp (a + b × T)) where M is the cumulative mortality, i and j referring to aphid and fungus species, respectively, parameter k is a maximal that M could reach during the bioassay, b is the intercept for the generated curve, and c the evolution rate of M (ij) over time (T). Since R-square values for non-linear models cannot be calculated directly, Efron’s pseudo R-squares were used [29]. One-way ANOVA coupled with a post-hoc Tukey test [30] were used to test for significant differences between the final maximum mortality for each treatment and to examine whether the non-linear least-square regression curves were significantly different. Median lethal time (LT_50_) for each tested aphid and fungus species combination was estimated using the invest () function from the “investor” R package [28]. T-tests were performed to compare the LT_50_ values.

## 3. Results

The mortality in the untreated controls was 13.3% (±5.8) for *S. avenae* and 16.7% (±5.8) for *R. padi* after nine days. The non-fungal mortality rate of *P. neoaphidis* exposed aphids (uncertain cause of death with no fungal symptom) was 12.0% (±8.4) for *S. avenae* and 14.0% (±5.5) for *R. padi* and non-fungal mortality of the *E. planchoniana* exposed aphids was 12.0% (±8.4) for *S. avenae* and 26.0% (±5.5) for *R. padi* (Supplementary Materials). We find significant differences in the overall mortalities over time between the different treatments (control, *P. neoaphidis* and *E. planchoniana*) (*p* < 0.0001) and aphid species (*p* < 0.0001) (Table 1).

The total numbers of fungus infections in the treated groups were as follows: after exposure to *P. neoaphidis* 80.0% (±7.1) of exposed *S. avenae* and 66.0 (±5.5) % of *R. padi* got infected; after exposure to *E. planchoniana* 68.0% (±8.4) of *S. avenae* and 48.0% (±11.0) of *R. padi* got infected. Significant differences were detected between the total numbers of fungus infections for each aphid species after treatments (Table 2).

Even though the ANOVA did not result in detection of an effect of the interaction between the two variables (fungi and aphid species), the post-hoc test detected significant differences between three specific combinations (Table 3). The total number of *R. padi* cadavers infected with *E. planchoniana* was significantly lower than that of *S. avenae* infected with the same fungus (*p* = 0.0107).

The time-mortality data *M (ij)* fitted well to the adopted non-linear estimation (Table 4) for *S. avenae* infected with *P. neoaphidis* (R^2^ = 0. 96) or *E. planchoniana* (R^2^ = 0.96) and fitted also for *R. padi* infected with *P. neoaphidis* (R^2^ = 0.97) or *E. planchoniana* (R^2^ = 0.93).

**Figure 2:**
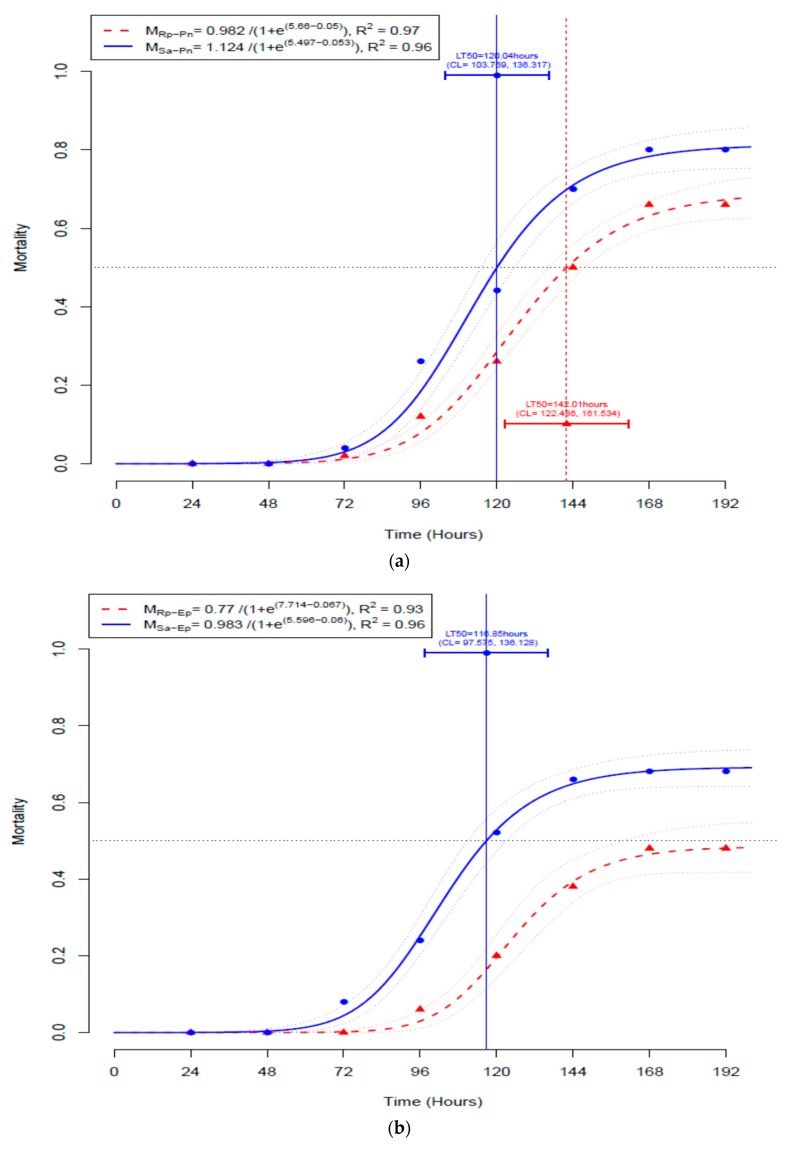
Time–mortality of fungus infected aphids. (**a**) *Sitobion avenae* (M_Sa-Pn_) and *Rhopalosiphum padi* (M_Rp-Pn_) infected with *Pandora neoaphidis*. (**b**) *Sitobion avenae* (M_Sa-Ep_) and *Rhopalosiphum padi* (M_Rp-Ep_) infected with *Entomophthora planchoniana*.

The proportion of mortality over time followed a sigmoid shape for the different treatments (Figure 2). 

Significant differences between the non-linear regression models were detected (Table 5), reflecting the differences between the aphid mortality from fungal treatments. In fact, the post-hoc Tukey test (Table 6) showed a significantly lower mortality rate of *R. padi* by *E. planchoniana* infection compared to the one registered for *S. avenae* either infected with *E. planchoniana* (*p* = 0.0032) or with *P. neoaphidis* (*p* = 0.0010). No significant difference was found between the mortality of *S. avenae* and *R. padi* over time after infection with *P. neoaphidis*.

LT_50_ estimates of *S. avenae* and *R. padi* infected with *P. neoaphidis* were 5.0 days (CL = 4.3, 5.7) and 5.9 days (CL = 5.1, 6.7), respectively. The estimated LT_50_ of *S. avenae* after *E. planchoniana* treatment was 4.9 days (CL = 4.1, 5.7). However, it was not possible to estimate LT_50_ for *R. padi* since the maximum mortality reached only 48.0% (±11.0) after the *E. planchoniana* treatment. The *t*-tests comparing the measured LT_50_ showed no significant differences between the values (Table 7).

## 4. Discussion

In this study, we mimicked the situation occurring in a natural agro-ecosystem and found a significant difference in aphid susceptibilities, possibly related to aphid host and/or fungus species and eventually aphid behavior. The conspecific host *S. avenae* was significantly more susceptible to *E. planchoniana* infection than the heterospecific host *R. padi*. However, we did not find any significant differences between the susceptibility of *R. padi* and *S. avenae* to *P. neoaphidis*. Differences in susceptibility between aphid species and morphs towards entomophthoralean fungal infection have been shown in several studies [2,31,32,33,34,35].

Our method simulates a real-life situation, where aphids were allowed to settle on the leaves before fungal treatments. Thus, some of the differences can potentially be attributed to the tendency of *R. padi* to settle lower on the plant than *S. avenae* does. By this behavioral resistance [36], it achieves partial protection from the infective conidia that “shower” from above. The measured differences in susceptibility between *R. padi* and *S. avenae* to *E. planchoniana* may reflect a general higher resistance in *R. padi* to specific fungal treatments, making this aphid less susceptible to the infection. Even though we did not consider the aphid microbiome composition in this study, the variation in susceptibility could be linked to diversity of facultative endosymbionts in *S. avenae* and *R. padi*. It has been shown previously, that bacterial symbionts can provide protection to their hosts from natural enemies [37]. Facultative endosymbionts (i.e., *Rigiella insecticola*, *Spiroplasma*, *Rickettsia*) have been reported to provide a significant protection to *Acyrthosiphon pisum* against *P. neoaphidis* [38,39] by reducing the mortality of their host and decreasing the fungal sporulation on the cadavers [38].

We did not find significant differences in the LT_50_ between either of the combinations of aphid or fungal species. The estimated LT_50_ values were within previously reported ranges for *P. neoaphidis* infection in both *S. avenae* and *R. padi* at 20 °C [40] and 17 °C [2], and for *E. planchoniana* infecting *A. fabae* [41]. Factors such as dose and incubation temperature can however influence the estimates [42]. In our bioassay, 66.0% of *R. padi* succumbed to *P. neoaphidis* infection, which was lower than reported previously [2].

In addition to counting the sporulating aphid cadavers, we also recorded the mortality of aphids with uncertain causes of deaths for each treatment. Interestingly, 26% of *R. padi* died with no visible fungal structures after treatment with *E. planchoniana*. Since we only used morphological tools to investigate infection, we cannot completely exclude fungal treatment as a cause of mortality. The relatively high mortality rate of *R. padi* could suggest a more specific host-pathogen relationship between this aphid and *E. planchoniana*. In fact, a previous study has reported a large genetic diversity among the fly-pathogenic Entomophthora compared to the relatively small aphid-pathogenic Entomophthora [43]. Such observations support the idea that host specialization might be an important factor in driving fungi within the genus *Entomophthora* and also the *E. planchoniana* virulence on aphids. The routine use of molecular techniques such as real-time qPCR [44] as additional means for detecting and quantifying the fungal pathogen in the dead aphids could further widen our knowledge on this highly structured interaction network.

The presence of *P. neoaphidis* or *E. planchoniana* infected aphid cadavers attached to a plant with healthy aphids could potentially enhance epizootic development [16]. Our results support this scenario, since both *P. neoaphidis* and *E. planchoniana* were able to infect both *S. avenae* and *R. padi* by conidia discharging from *S. avenae* cadavers. The coexistence of healthy and infected aphids occurring on the same host plant allows the transmission and establishment of fungal infections between conspecific and heterospecific aphids, in the latter case potentially with more resistance. Such observations might push forward attempts [45] to use entomophthoralean fungi as an effective biological control agent over insect pests.

## 5. Conclusions

We standardized a methodology to allow a direct comparison of entomophthoralean fungal virulence against two aphid hosts. The transmission model designed in this study shows a successful in vivo establishment of the infection by two specialized aphid pathogens, *P. neoaphidis* and *E. planchoniana*, in conspecific and heterospecific aphids; which should be implemented in future biological control programs against aphid pests in cereals.

## Figures and Tables

**Table 1:** Results of ANOVA analysis comparing the overall mortalities over time for each aphid species for the adopted treatments. The interactions between aphid species, treatments and aphid species were also analyzed. Abbreviations: *df* = degree of freedom, *SumSq* = sum of squares, *MeanSq* = mean of squares, *F* = test value, *p* = significance level. Values in bold indicate significant differences (*p* ≤0.05).

	ANOVA				
	*SumSq*	*Df*	*MeanSq*	*F*	*p*
Time (T)	14.239	1	14.239	1388.45	**<0.0001**
Treatments (R)	14.295	2	7.148	696.98	**<0.0001**
Aphid species (A)	0.661	1	0.661	64.452	**<0.0001**
T*R	8.688	2	4.344	423.575	**<0.0001**
T*A	0.117	1	0.117	11.441	**0.0008**
R*A	0.463	2	0.232	22.59	**<0.0001**
T*R*A	0.071	2	0.035	3.451	0.0332
Residuals	2.502	244	0.010		

**Table 2:** Results of ANOVA analysis comparing the total numbers of fungus infections values for each aphid species after infection by *P. neoaphidis* and *E. planchoniana*. The interaction between aphid species and fungus species was also analyzed. Abbreviations: *df* = degree of freedom, *SumSq* = sum of squares, *MeanSq* = mean of squares, *F* = test value, *p* = significance level. Values in bold indicate significant differences (*p* ≤ 0.05).

	ANOVA				
	*SumSq*	*df*	*MeanSq*	*F*	*p*
Fungus species (F)	0.131	1	0.131	16.13	**0.0001**
Aphid species (A)	0.172	1	0.172	21.07	**0.0003**
F*A	0.002	1	0.002	0.30	0.587
Residuals	0.130	16	0.008		

**Table 3:** Results of Post-hoc Tukey test for multiple comparisons of means with 95% family-wise confidence level. Abbreviations: *diff* = difference between group means, *lower* = lower end point of the interval, *upper* = upper end point of the interval, *p* = significance level. Values in bold indicate significant differences (*p* ≤ 0.05) between the different combinations of the treatment and aphid host species.

		Post-hoc Tukey Test			
Combination 1	Combination 2	*Diff*	*Lower*	*Upper*	*p*
*P. neoaphidis*-*R. padi*	*E. planchoniana*-*R. padi*	0.184	0.021	0.348	**0.0100**
*E. planchoniana*-*S. avenae*	*E. planchoniana*-*R. padi*	0.207	0.044	0.371	**0.0107**
*P. neoaphidis*-*S. avenae*	*E. planchioniana*-*R. padi*	0.347	0.184	0.511	**<0.0001**
*E. planchoniana*-*S. avenae*	*P. neoaphidis*-*R. padi*	0.023	−0.140	0.186	0.9766
*P. neoaphidis*-*S. avenae*	*P. neoaphidis*-*R. padi*	0.163	−0.0003	0.326	0.0506
*P. neoaphidis*-*S. avenae*	*E. planchoniana*-*S. avenae*	0.140	−0.023	0.303	0.1074

**Table 4:** Estimation of the non-linear least-square regression model of the mortality of *Sitobion avenae* and *Rhopalosiphum padi* to fungal infection.

Fungus Species	Aphid Species	Estimated Parameters ^a^ and Fitness						
		K (SD)	*t*-Values	a (SD)	*t*-Values	b (SD)	*t*-Values	R^2^
*P. neoaphidis*	*S. avenae*	1.124 (±0.036)	31.42	5.497 (±0.591)	9.30	−0.053 (±0.006)	−8.66	0.96
	*R. padi*	0.982 (±0.033)	29.86	5.66 (±0.534)	10.59	−0.05 (±0.005)	−9.63	0.97
*E. planchonian* *a*	*S. avenae*	0.983 (±0.026)	37.31	5.596 (±0.63)	8.88	−0.06 (±0.007)	−8.51	0.96
	*R. padi*	0.77 (±0.034)	22.66	7.714 (±1.332)	5.79	−0.067 (±0.012)	−5.56	0.93

^a^ A non-linear regression: *M (ij) = k/(1 + exp (a + b × T))*.

**Table 5:** Results of ANOVA analysis comparing the designed non-linear least-square regression models for each treatment. Abbreviations: *df* = degree of freedom, *SumSq* = sum of squares, *MeanSq* = mean of squares, *F* = test value, *p* = significance level. Values in bold indicate significant interactions (*p* ≤ 0.05).

	ANOVA				
	*SumSq*	*df*	*MeanSq*	*F*	*p*
Models	2.81	3	0.935	6.15	**0.0004**
Residuals	60.25	396	0.152		

**Table 6:** Results of post-hoc Tukey test for multiple comparisons of means with 95% family-wise confidence level. Abbreviations: *diff* = difference between group means, *lower* = lower end point of the interval, *upper* = upper end point of the interval, *p* = significance level. Values in bold indicate significant differences (*p* ≤ 0.05) between the different non-linear regression models.

		*Post-hoc Tukey Test*			
Model 1	Model 2	*Diff*	*Lower*	*Upper*	*p*
*P. neoaphidis-R. padi*	*E. planchoniana-R. padi*	0.093	−0.049	0.235	0.3318
*E. planchoniana-S. avenae*	*E. planchoniana-R. padi*	0.191	0.048	0.333	**0.0032**
*P. neoaphidis-S. avenae*	*E. planchioniana-R. padi*	0.208	0.066	0.351	**0.0010**
*E. planchoniana-S. avenae*	*P. neoaphidis-R. padi*	−0.098	−0.240	0.044	0.2839
*P. neoaphidis-S. avenae*	*P. neoaphidis-R. padi*	0.115	−0.026	0.258	0.1547
*P. neoaphidis-S. avenae*	*E. planchoniana-S. avenae*	0.017	−0.124	0.159	0.9887

**Table 7:** Results of t-tests for LT_50_ comparisons. Abbreviations: ne = non estimated values due to the absence of LT_50_ related to the mortality of *R. padi* after treatment with *E. planchoniana*.

Model 1	Model 2	*t*-Values
*E. planchoniana-R. padi*	*E. planchoniana-S. avenae*	ne
*E. planchoniana-R. padi*	*P. neoaphidis-R. padi*	ne
*E. planchoniana-R. padi*	*P. neoaphidis-S. avenae*	ne
*E. planchoniana-S. avenae*	*P. neoaphidis-R. padi*	0.050
*E. planchoniana-S. avenae*	*P. neoaphidis-S. avenae*	0.402
*P. neoaphidis-R. padi*	*P. neoaphidis-S. avenae*	0.941

## Supplementary Materials

The following are available online at http://www.mdpi.com/2075-4450/10/2/54/s1, Table S1: Aphid-mortality records over time (hours), after the two fungal treatments, for each aphid species. Replicates, number of incubated aphids, total numbers of dead aphids are presented. Total numbers of dead aphids, is split to deaths caused by fungus and those did not show any fungal infection symptom, and therefore, the cause of death could not be determined. Script S1: R code for analyzing the data including statistical tests and plotting.

## References

[1] Blackman R.L., Eastop V.F., Van Emden H.F., Harrington R. (2007). Taxonomic Issues. Aphids as Crop Pests.

[2] Nielsen C., Steenberg T. (2004). Entomophthoralean fungi infecting the bird cherry-oat aphid, *Rhopalosiphum padi*, feeding on its winter host bird cherry, *Prunus padus*. J. Invertebr. Pathol..

[3] Dedryver C.A., Le Ralec A., Fabre F. (2010). The conflicting relationships between aphids and men: A review of aphid damage and control strategies. C. R. Biol..

[4] Barta M., Cagan L. (2006). Aphids-pathogenic Entomophthorales (their taxonomy, biology and ecology). Biologia.

[5] Humber R.A. (2012). Entomophthoromycota: A new phylum and reclassification for entomophthoroid fungi. Mycotaxon.

[6] Ben Fekih I., Boukhris-Bouhachem S., Eilenberg J., Allagui M.B., Jensen A.B. (2013). The Occurrence of two species of Entomophthorales (Entomophthoromycota), pathogens of *Sitobion avenae* and *Myzus persicae* (Hemiptera: Aphididae), in Tunisia. Biomed. Res. Int..

[7] Manfrino R.G., Gutierrez A.C., Steinkraus D.C., Salto C.E., Lopez Lastra C.C. (2014). Prevalence of entomophthoralean fungi (Entomophthoromycota) of aphids (Hemiptera: Aphididae) on solanaceous crops in Argentina. J. Invertebr. Pathol..

[8] Lacey L.A., Frutos R., Kaya H.K., Vail P. (2001). Insect pathogens as biological control agents: Do they have a future?. Biol. Control.

[9] Scorsetti A.C., Humber R.A., Garcia J.J., Lastra C.C.L. (2007). Natural occurrence of entomopathogenic fungi (Zygomycetes: Entomophthorales) of aphid (Hemiptera: Aphididae) pests of horticultural crops in Argentina. Biocontrol.

[10] Ben Fekih I., Boukhris-Bouhachem S., Allagui M.B., Jensen A.B., Eilenberg J. (2015). First survey on ecological host range of aphid pathogenic fungi (Phylum Entomophthoromycota) in Tunisia. Ann. Soc. Entomol. Fr. (NS).

[11] Papierok B., Dedryver C.A., Hullé M. (2016). First records of aphid-pathogenic Entomophthorales in the sub-Antarctic archipelagos of Crozet and Kerguelen. Polar Res..

[12] Fournier A. (2010). Assessing Winter Survival of the Aphid Pathogenic Fungus *Pandora neoaphidis* and Implications for Conservation Biological Control. Ph.D. Thesis.

[13] Lacey L.A. (2012). Manual of Techniques in Invertebrate Pathology.

[14] Eilenberg J., Bresciani J., Olesen U., Olson L. (1995). Ultrastructural studies of secondary spore formation and discharge in the genus *Entomophthora*. J. Invertebr. Pathol..

[15] Roy H.E., Steinkraus D., Eilenberg J., Hajek A.E., Pell J.K. (2006). Bizarre interactions and endgames: Entomopathogenic fungi and their arthropod hosts. Annu. Rev. Entomol..

[16] Steinkraus D.C. (2006). Factors affecting transmission of fungal pathogens of aphids. J. Invertebr. Pathol..

[17] Lacey L.A., Grzywacz D., Shapiro-Ilan D.I., Frutos R., Brownbridge M., Goettel M.S. (2015). Insect pathogens as biological control agents: Back to the future. J. Invertebr. Pathol..

[18] Pell J.K., Eilenberg J., Hajek A.E., Steinkraus D.C., Butt T.M., Jackson C.W., Magan N. (2001). Biology, ecology and pest management potential of Entomophthorales. Fungi as Biocontrol Agents. Progress, Problems and Potential.

[19] Hemmati F., Pell J.K., McCartney H.A., Deadman M.L. (2002). Aerodynamic diameter of conidia of *Erynia neoaphidis* and other entomophthoralean fungi. Mycol. Res..

[20] Pell J.K., Ekesi S., Manianai N. (2007). Ecological approaches to pest management using entomopathogenic fungi: Concepts, theory, practice, and opportunities. Use of Entomopathogenic Fungi in Pest Management.

[21] Hajek A.E., Delalibera I.J. (2010). Fungal pathogens as classical biological control agents against arthropods. BioControl.

[22] Hajek A.E., Papierok B., Eilenberg J., Lacey L.A. (2012). Methods for study of the Entomophthorales. Manual of Techniques in Invertebrate Pathology.

[23] Shah P.A., Clark S.J., Pell J.K. (2003). Direct and indirect estimates of *Pandora neopahidis* conidia in laboratory bioassays with aphids. J. Invertebr. Pathol..

[24] Humber R.A., Lacey L.A. (1997). Fungi: Identification. Manual of Techniques in Insect Pathology.

[25] Freeman M.F., Tukey J.W. (1950). Transformations related to the angular and the square root. Ann. Math. Stat..

[26] R Core Team (2012). R: A Language and Environment for Statistical Computing. https://www.r-project.org/.

[27] Dowle M., Srinivasan A. (2018). Data.Tableable: Extension of ‘Data.Frame’. https://CRAN.R-project.org/package=data.table.

[28] Greenwell B.M., Kabban C.M.S. (2014). Investr: An R Package for Inverse Estimation. Version 1.4.0. R J..

[29] Scott Long J., Freese J. (2014). Regression Models for Categorical Dependent Variables Using Stata.

[30] Tukey J. (1949). Comparing Individual Means in the Analysis of Variance. Biometrics.

[31] Dromph K.M., Pell J.K., Eilenberg J. (2002). Influence of flight and colour morph on susceptibility of *Sitobion avenae* to infection by *Erynia neoaphidis*. BioControl Sci. Technol..

[32] Shah P.A., Clark S.J., Pell J.K. (2004). Assessment of aphid host range and isolate variability in *Pandora neoaphidis* (Zygomycetes: Entomophthorales). Biol. Control.

[33] Xu J.H., Feng M.G. (2000). The time–dose–mortality modeling and virulence indices for two entomophthoralean Species, *Pandora delphacis* and *P. neoaphidis*, against the green peach aphid, *Myzus persicae*. Biol. Control.

[34] Ekesi S., Shah P.A., Clark S.J., Pell J.K. (2005). Conservation biological control with the fungal pathogen *Pandora neoaphidis*: Implications of aphid species, host plant and predator foraging. Agric. Forest. Entomol..

[35] Hesketh H., Alderson P.G., Pye B.J., Pell J.K. (2008). The development and multiple uses of a standardised bioassay method to select hypocrealean fungi for biological control of aphids. Biol. Control.

[36] Jensen S.E. (2000). Insecticide resistance in the western flower thrips, *Frankliniella occidentalis*. Integr. Pest Manag. Rev..

[37] Moran N.A., McCutcheon J.P., Nakabachi A. (2008). Genomics and evolution of heritable bacterial symbionts. Annu. Rev. Genet..

[38] Łukasik P., Guo H., Van Asch M., Ferrari J., Godfray H.C.J. (2013). Protection against a fungal pathogen conferred by the aphid facultative endosymbionts *Rickettsia* and *Spiroplasma* is expressed in multiple host genotypes and species and is not influenced by co-infection with another symbiont. J. Evol. Biol..

[39] Heyworth E.R., Ferrari J. (2015). A facultative endosymbiont in aphids can provide diverse ecological benefits. J. Evol. Biol..

[40] Nielsen C., Eilenberg J., Dromph K. (2001). Entomophthorales on Cereal Aphids: Characterisation, Growth, Virulence, Epizootiology and Potential for Microbial Control.

[41] Brobyn P., Wilding N. (1977). Invasive and developmental processes of *Entomophthora* species infecting aphids. Trans. Br. Mycol. Soc..

[42] Feng M.G., Poprawski T.J., Nowierski R.M., Zeng Z. (1999). Infectivity of *Pandora neoaphidis* (Zygomycetes: Entomophthorales) to *Acyrthosiphon pisum* (Hom., Aphididae) in response to varying temperature and photoperiod regimes. J. Appl. Entomol..

[43] Jensen A.B., Eilenberg J., Lastra C.L. (2009). Differentially aphid and fly host driven divergence of obligate insect pathogenic Entomophthora species. FEMS Microbiol. Lett..

[44] Chen C., Ye S., Hu H., Xue C., Yu X. (2018). Use of electrical penetration graphs (EPG) and quantitative PCR to evaluate the relationship between feeding behaviour and *Pandora neoaphidis* infection levels in green peach aphid, *Myzus persicae*. J. Insect Physiol..

[45] Dinu M.M., Bloemhard C.M.J., van Holstein-Saj R., Messelink G.J. (2017). Exploring opportunities to induce epizootics in greenhouse aphid populations. Acta Hortic..

